# Attention Towards Pupil Size in Humans and Bonobos (*Pan paniscus*)

**DOI:** 10.1007/s42761-022-00146-1

**Published:** 2022-11-11

**Authors:** T. W. Zijlstra, E. van Berlo, M. E. Kret

**Affiliations:** 1 Cognitive Psychology Unit, Institute of Psychology, Leiden University, Leiden, the Netherlands; 2 Leiden Institute for Brain and Cognition (LIBC), Leiden, the Netherlands; 3 Institute for Biodiversity and Ecosystem Dynamics, University of Amsterdam, Amsterdam, the Netherlands

**Keywords:** Pupil, Attention, Eyes, Imitation, Bonobos, Sociality

## Abstract

Previous work has established that humans have an attentional bias towards emotional signals, and there is some evidence that this phenomenon is shared with bonobos, our closest relatives. Although many emotional signals are explicit and overt, implicit cues such as pupil size also contain emotional information for observers. Pupil size can impact social judgment and foster trust and social support, and is automatically mimicked, suggesting a communicative role. While an attentional bias towards more obvious emotional expressions has been shown, it is unclear whether this also extends to a more subtle implicit cue, like changes in pupil size. Therefore, the current study investigated whether attention is biased towards pupils of differing sizes in humans and bonobos. A total of 150 human participants (141 female), with a mean age of 19.13 (ranging from 18 to 32 years old), completed an online dot-probe task. Four female bonobos (6 to 17 years old) completed the dot-probe task presented via a touch screen. We used linear mixed multilevel models to examine the effect of pupil size on reaction times. In humans, our analysis showed a small but significant attentional bias towards dilated pupils compared to intermediate-sized pupils and intermediate-sized pupils when compared to small pupils. Our analysis did not show a significant effect in bonobos. These results suggest that the attentional bias towards emotions in humans can be extended to a subtle unconsciously produced signal, namely changes in pupil size. Due to methodological differences between the two experiments, more research is needed before drawing a conclusion regarding bonobos.

**Supplementary Information:**

The online version contains supplementary material available at 10.1007/s42761-022-00146-1.

For social animals such as humans and bonobos, it is essential to estimate a group member’s emotional state accurately and quickly (Darwin, 1872; LeDoux, 1995; Kret et al., 2020). One trait that enables individuals to respond appropriately is an attentional bias towards emotional signals (Phelps et al., 2006; Schupp et al., 2003; Vuilleumier, 2005; van Rooijen et al., 2017). Several studies suggest that subtle signals, like changes in pupil size, may also play an important communicative role (Kret, 2015). However, whether an attentional bias towards emotional cues extends to these subtle signals is still unknown. Moreover, given that emotional expressions also receive prioritized attention in some other, non-human primates (Kret et al., 2016; Lacreuse et al., 2013; Parr et al., 2013), an important question is whether a potential bias towards pupil size changes is uniquely human or a derived trait shared with other species. In the current study, we therefore investigated whether humans and bonobos have an attentional bias towards conspecifics’ pupil size in a dot-probe task.

Emotional expressions can communicate several emotion states and help to predict the course of social interactions (Cosmides & Tooby, 2000; Liebal et al., 2013). While an angry expression of a group member may reflect a threat, potentially causing conflict, a happy expression might represent social opportunities. The correct recognition of emotional cues of group members therefore has benefits, and therewith, great survival value (Darwin, 1872; Spoor & Kelly, 2004; de Waal, 2008). Although many emotional signals are explicit and overt, there are also implicit cues that contain emotional information, such as facial reddening, tears, and changes in pupil size. While these signals tend to be more subtle, they seem sufficient to foster trust and social support (Kret, 2015).

Eye contact may provide a powerful mode of sharing emotions through subtle expressions. Eyes not only enable an individual to see the world around them but also serve as a reference point for other individuals (Silk et al., 2008). Moreover, when zooming in on the eyes, the pupils provide a subtle window into an individual’s emotional state. Although one primary function of the pupil is to dilate or constrict in response to light, it can also reflect autonomic responses originating from within the individual. For instance, in several studies, participants showed pupil dilation in response to viewing stimuli with emotionally relevant content (Lester & Gatto, 1990; Muris et al., 2001; Kret, Roelofs et al., 2013, Kret, Stekelenburg et al., 2013), and this phenomenon already occurs in infancy (Geangu et al., 2011; Fawcett et al., 2017; Aktar et al., 2020). Furthermore, the dilation of the pupils may reflect a variety of socially relevant signals, such as social interest and attention, surprise or uncertainty, and arousal in combination with several emotions (Bradshaw, 1967; Hess, 1975; Lavín et al., 2014). Conversely, pupil constriction is associated with fatigue and boredom (Lowenstein et al., 1963). Additionally, pupil size changes temporally align with the moment individuals make a decision about the nature of a social signal, suggesting a link between pupil changes and emotion recognition (Oliva & Anikin, 2018).

In addition to reflecting inner states, changes in pupil size appear to have a social signalling function (Quesque et al., 2019) and can be considered a form of basic communication between conspecifics (Kret et al., 2014). Investigating the social value of changes in pupil size, Hess (1975) established that women with larger pupils were described by men as “more feminine,” “prettier,” and “softer” compared to women with small pupils (Hess, 1975). Individuals with large pupils tend to be viewed as more positive and attractive, while individuals with small pupils are judged to be cold and distant (Demos et al., 2008; Amemiya & Ohtomo, 2012). Larger pupils were also found to be associated with increased approach behavior and attractiveness in humans (Clark et al., 2013; Brambilla et al., 2019).

While pupillary changes in others can be detected by observers when instructed to do so, pupil sizes are typically perceived and produced unconsciously during social interactions (Harrison et al., 2009; Bradshaw, 1967). This can have important implications for communication, because while explicit signals such as certain emotional expressions can be culture specific and can be controlled (Schmidt & Cohn, 2001), the implicit and unconscious reactions to changes in pupil size are likely universally shared (Kret et al., 2014), providing an honest signal. As such, automatically detecting changes in another’s pupils can help the observer acquire relevant information about their partner’s emotional or mental state (Kret & Ploeger, 2015).

Various studies in humans have shown that at the earliest stages of processing information, attention tends to be biased towards stimuli with emotional content when compared to emotionally neutral stimuli (Vuilleumier & Schwartz, 2001; Vuilleumier, 2005; Lang et al., 1998; van Rooijen et al., 2017). This bias towards emotional information is likely the result of a fast and automatic mechanism that helps individuals to respond quickly but also adequately to threatening situations (Öhman et al., 2001; Frijda, 2010; LeDoux, 1995).

Moreover, this bias towards emotions is likely not uniquely human, and findings suggest it is at least shared with bonobos (*Pan paniscus*), our closest living relatives. For instance, Kret et al. (2016) observed that bonobos (*Pan paniscus*) have an attentional bias towards (mainly positive) emotional cues such as scenes involving sexual behavior, yawning, and grooming. In line with these findings on prioritized processing of emotional signals, other works suggest a sensitivity to emotions in bonobos. Laméris et al. (2022) found that bonobos’ performance on an emotional Stroop task was affected by seeing play faces, also considered a positively valenced social signal (Palagi, 2008). Moreover, in an eye-tracking study, bonobos made more eye contact when compared to chimpanzees (*Pan troglodytes*), our other closest relatives (Kano et al., 2015). This finding suggests that an ability to properly evaluate facial expressions and pay attention to them may be of great importance to bonobos, similar to humans (Farroni et al., 2002).

Currently, no work has investigated whether subtle signals such as changes in pupil size receive attentional priority. Given a shared attentional bias for emotional signals in humans and bonobos, and a similar propensity to make eye contact, bonobos are an ideal model to study a potential bias for pupillary changes from a comparative perspective. One experimental paradigm that has been used successfully to investigate implicit attentional biases is the dot-probe task (van Rooijen et al., 2017). Since the dot-probe task requires little training or explanation, and allows us to present an experiment with a very similar procedure to both humans and bonobos, this task is ideally suited to study attentional biases from a comparative perspective. Previous dot-probe studies have shown a bias in spatial attention by comparing reaction times on trials in which a dot was presented at the location of a previously presented emotional stimulus when compared to trials in which the dot was presented at the location of a neutral stimulus. (Williams et al., 1996; Bradley et al., 1997; Carlson & Reinke, 2008, Holmes et al., 2005; de Valk et al., 2015). Lastly, the dot-probe has also been used successfully to investigate attentional biases in bonobos and other great apes (e.g., Kret et al., 2016; Kret et al., 2018; Tomonaga & Imura, 2009). Although an attentional bias towards explicit emotional signals and scenes has been established, it is unclear whether this can be generalized to more subtle signals like changes in pupil size.

We therefore conducted two dot-probe experiments, one in humans and one in bonobos, to answer whether they had an attentional bias towards alterations in pupil size. More specifically, the experiments investigated whether humans’ and bonobos’ attention is modulated by differences in pupil size in the eyes of conspecifics. Specifically, we expected that large pupils would attract attention in humans when compared to intermediate-sized pupils, since dilated pupils may be indicative of an individual’s increased level of arousal. Because constricted pupils are associated with fatigue or boredom (Lowenstein et al., 1963) and observers perceive individuals with smaller pupil sizes more negatively (Demos et al., 2008; Amemiya & Ohtomo, 2012), we hypothesized that there might also be an attentional bias towards small pupils when compared to intermediate-sized pupils. In our bonobo sample, we compared small and large pupil sizes directly. Because aggression in bonobo society is usually prevented through nonreproductive sexual behavior (de Waal, 1990; Hare et al., 2012, Tan & Hare, 2013) and bonobos have shown the strongest bias towards positive emotional scenes (Kret et al., 2016), we expected them to have a bias towards positive signals compared to negative signals. We therefore hypothesized that they would show an attentional bias towards large compared to small pupil sizes.

## Experiment 1: Humans

### Method: Experiment 1

#### Participants

A total of 150 participants (141 female) with a mean age of 19.1 (ranging from 18 to 32 years old) completed an online dot-probe task. The task was presented via the online platform “Gorilla” and all participants completed the task between 01-10-2020 and 24-12-2020. Participants were recruited via the online recruitment platform SONA of Leiden University, as well as via an advertisement on Facebook. Psychology students from Leiden University could earn 1 study credit by completing the task. One participant was excluded because more than 50% of their trials did not meet our criteria for inclusion. All participants gave their informed consent before taking part in the experiment. The study was approved by the Psychology Research Ethics Committee of Leiden University (2020-08-25-M.E.Kret-V2-2570).

#### Stimuli

We presented stimuli consisting of color images of the eye region of 22 different Caucasian individuals (11 female). The pupil size in these images had been manipulated to create 3 different images of the same individual (with small, intermediate-sized, and large pupils that had diameters of 3mm, 5mm, and 7mm respectively). Mean and standard deviation of luminance as well as RMS contrast were calculated in MATLAB and manually adjusted when necessary to ensure similar luminance between pairs of stimuli (see supplements (Table ESM4) for an overview of luminance ratings). Image manipulation was done in Photoshop CC. Stimuli were sized 700 by 277 pixels.

#### Procedure

Before the start of the experiments, participants were informed about the content and goal of the study as well as the procedure. They were informed about how their data would be protected, handled, and stored and that their participation was voluntary and could be stopped at any moment. All participants provided written consent for participating in this study and for the use of their data for analyses. After consenting, the participant was asked to provide their age and sex. Next, participants received information about the procedure of the task, namely what keys to use during the experiment, to turn off or silence their telephones to avoid distractions, and to maximize their browser window. Finally the participant was asked to press the spacebar to start the experiment.

**Fig. 1:**
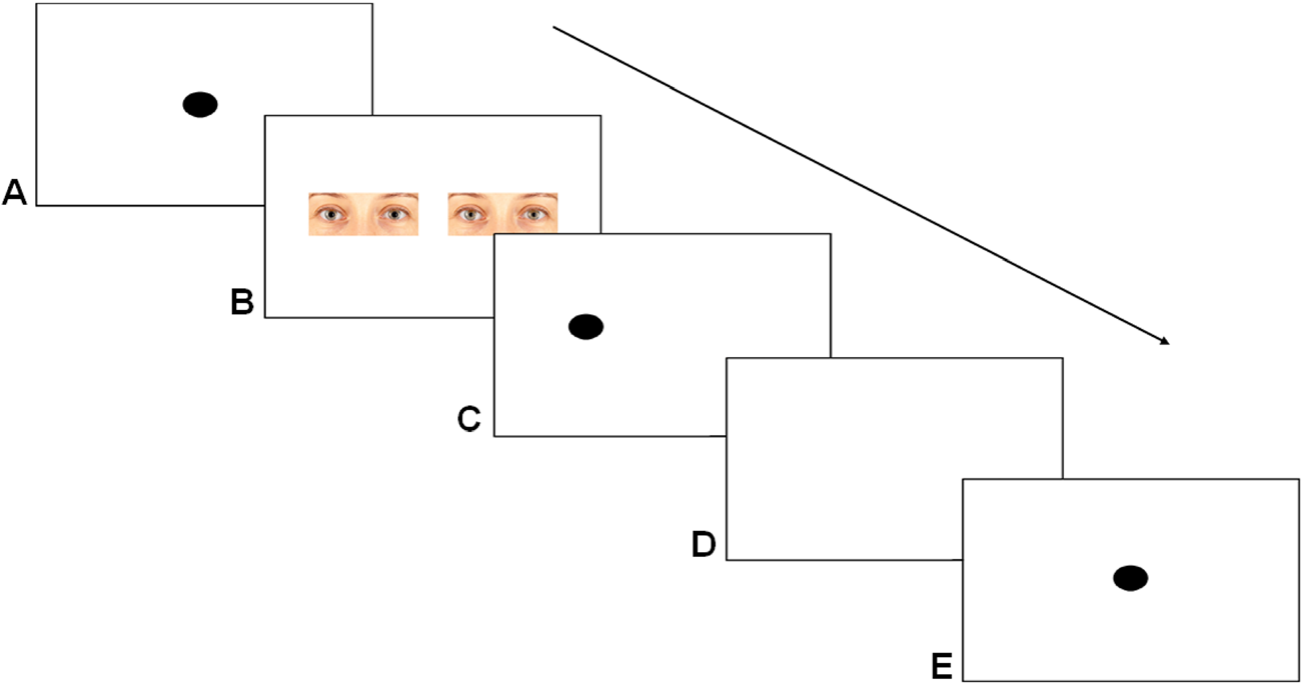
A trial outline. In each trial, a dot is presented (**A**; in the human experiment, this was replaced with a fixation cross), after which the stimuli were presented for 300 ms (**B**). The probe replaced either the right or the left image (**C**). Following a response from the participant, a blank screen was presented during the inter-trial interval (**D**; 200 ms for humans and 2,000ms for bonobos). After the inter-trial interval, a new trial is started and the same procedure is repeated (**E**)

All participants performed the same dot-probe task, consisting of the same stimulus set. Each trial started with a fixation cross (presented for 500 ms), followed by two stimuli (presented for 300 ms), showing the same individual but with different pupil sizes presented side-by-side in two different combinations (small vs. intermediate-sized and large vs. intermediate-sized). After presentation of the stimuli, a black dot (probe) appeared in the position of one of the two stimuli. Participants were asked to indicate on which side the probe appeared by pressing the corresponding keyboard key (“f” for left and “j” for right). After pressing either of the two keys, a blank screen was shown for an inter-trial interval of 200 ms after which the next trial started (see Fig. 1 for a trial outline). Each participant performed three practice trials that included pictures of animals (a duck, a goose, and a sheep), after which the task was started. After 88 trials, the participant was able to take a short break, and could continue with the rest of the experiment by pressing the spacebar. Individuals were shown all 22 stimuli in the two combinations and each combination was shown four times (twice before a short break, twice after), resulting in a total number of 176 trials. The order of trials was randomized and both the position of the two images and the position at which the second dot appeared were counter balanced between left and right.

#### Statistical Analysis

Following a 2×2 factorial design, we used linear mixed multilevel models to examine the effects of stimulus pupil size (Large/Small) and congruence (Congruent/incongruent) on reaction times. Pupil size was coded as “Large” when large and intermediate-sized pupils were shown and coded as “Small” when small and intermediate-sized pupils were shown, while the dot position (hereafter called “Congruency”) was coded as “Congruent” when the dot appeared in the position of the large or small pupils and as “Incongruent” when the dot appeared in the location of the intermediate-sized pupils (i.e., the controls). Trials were nested within subjects. We added random intercepts for subjects, for stimulus and for “order of trials.” This resulted in the model: Reaction time ~ Congruency * Pupil size + (1|subject)+(1|stimulus)+(1|trial). This model and a null model (that only included the random effects) were subsequently compared with each other based on AIC scores (Akaike, 1974; Symonds & Moussalli, 2011).

All trials in which an incorrect response was given (i.e., when the participant pressed the wrong key, such as the left key “f” when a probe appeared on the right side) were excluded. All reaction times below 200 ms and above 5000 ms were excluded by default. Subsequently, all reaction times that deviated from the mean by more than 2.5 times the median absolute deviation (MAD) were excluded (Leys et al., 2013). This MAD filtering was done per participant. Any participant for whom more than 50% of observations were outside of these thresholds was excluded from the experiment. As a result, 1 (female) participant was excluded. See Table 1 for an overview of the number of included trials.

**Table 1:** Number of included trials: Experiment 1

Comparison	Large - Intermediate		Small - Intermediate	
Congruency	Congruent	Incongruent	Congruent	Incongruent
Number of included trials per participant	Mean: 39.7	Mean: 39.1	Mean: 39.4	Mean: 39.8
	Min: 23	Min: 21	Min: 23	Min: 19
	Max: 44	Max: 44	Max: 44	Max: 44
Percentage of trials excluded per participant	Mean: 9.7	Mean: 11.1	Mean: 10.5	Mean: 9.5
	Min: 0	Min: 0	Min: 0	Min: 0
	Max: 47.7	Max: 52.3	Max: 47.7	Max: 56.8

The statistical analysis was performed in R (R Core Team, 2020), using the “lme4” package (Bates et al., 2015).

### Results: Experiment 1

To investigate an attentional bias towards pupil size in humans, we constructed a multilevel model containing congruency, pupil size, and the potential interaction between these two factors, as fixed effect, as well as subject, trial number, and stimulus as random effects. This model was a significantly better fit than the null model that contained the same random effects but not the fixed effects (delta AIC = 12). See supplements (Table ESM1) for an overview of the model comparison.

**Fig. 2:**
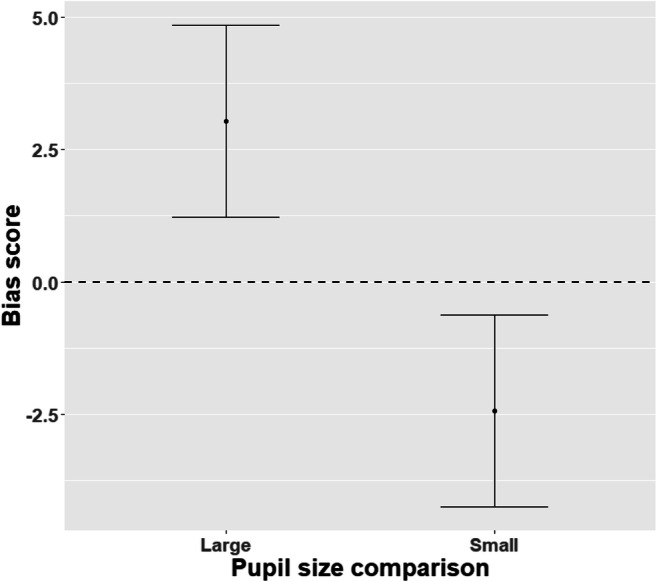
Bias score for large and small pupil sizes. The *y*-axis shows the difference in reaction time between congruent and incongruent trials for both large pupils compared to intermediate-sized pupils (left) and small pupils compared to intermediate-sized pupils (right). A larger bias score reflects that reaction times in the congruent trials were faster. Bias scores are in milliseconds. The bars indicate a 95% confidence interval around the estimate

The analysis showed a small but significant effect of the interaction between congruency and pupil size on reaction times (*t* = −4.201, *p* < 0.001), as well as a significant effect of pupil size (*t* = 3.380, *p* < 0.001) and congruency (*t* = 3.290, *p* = 0.001; see Table 2 for an overview of these results). A post hoc pairwise comparison was performed to further investigate this effect (see Fig. 2). When large and intermediate-sized pupils were presented, reaction times were significantly faster in trials where the dot replaced large pupils (−3.04 ms, SE = 0.925, *z* = −3.290, *p* = 0.001). When intermediate-sized pupils were presented next to small pupils, reaction times were significantly faster in trials where the dot replaced the intermediate-sized pupils (2.44 ms, SE = 0.923, *z* = 2.649, *p* = 0.008).

**Table 2:** Results on the analysis on the effect of interaction between congruency and pupil size on reaction times

Fixed effects	Estimate	Std. error	*T* value	*p*	Cohen’s *d*
Pupil size (small)	3.1203	0.9232	3.380	<0.001	0.046
Congruency (incongruent)	3.0434	0.9249	3.290	0.001	0.045
Pupil size*Congruency	−5.4878	1.3064	−4.201	<0.001	0.080

### Conclusion: Experiment 1

In this experiment, humans displayed a small but significant bias towards larger pupils when compared to smaller pupils. This was true for trials in which large pupils were compared to intermediate-sized pupils, as well as for trials in which intermediate-sized pupils were compared to small pupils.

## Experiment 2: Bonobos

### Method: Experiment 2

#### Participants

Four female bonobos (*Pan paniscus*, aged between 6 and 17 years old) participated in this study. The bonobos were housed at Primate park Apenheul in the Netherlands and were living in a social group consisting of 12 individuals (8 females, 4 males). Individuals participated voluntarily and were never separated from their group members. Testing took place when the park was closed off to visitors (in the winter). Bonobos were tested two to four times a week inside their indoor enclosure, with test sessions lasting between 15 and 20 min. Before the start of this study, bonobos were extensively trained to work on a touchscreen (see, e.g., Kret et al., 2016 for details on training).

The study was conducted adhering to the guidelines of the EAZA Ex-situ Program (EEP), formulated by the European Association of Zoos and Aquaria (EAZA). To engage the bonobos in the study, only positive reinforcement was used in the form of a food reward (apple cubes). Bonobos always had access to water and were fed regularly throughout the day. Moreover, non-participating bonobos received rewards equivalent to the participating bonobos to ensure group stability and prevent stressful situations.

#### Stimuli

We presented stimuli consisting of color images of the face of individuals living in the same group as the participating bonobos (*n* = 168) and of unfamiliar individuals from other zoos (*n* = 50) to maximize the number of unique stimuli in our dataset. The stimuli differed somewhat from Experiment 1. Firstly, we used portrait images of the bonobos rather than just the eye region. Secondly, there was some variation between head position and gaze direction of the individual in the pictures (see supplements for examples: Figure ESM1; also see Table ESM5 for an overview of luminance ratings). We chose these images because standardized images of faces and eye regions of bonobos (looking directly at the camera) were not available. However, both eyes were always clearly visible in all pictures. For each of the 12 individuals living in the group, we obtained two to nine unique pictures, and another 25 unique pictures from unfamiliar bonobos taken from the internet. For each of these pictures, we manipulated the pupils to be small (6 pixels) and large (8 pixels), resulting in a total of 218 stimuli (i.e., 109 unique stimuli, manipulated to have small and large pupils). Due to time constraints in testing the bonobos, we opted to only compare small to large pupils and leave out the intermediate pupil size condition.

#### Apparatus

The study was conducted using the software Presentation (NeuroBehavioralSystems) and a touchscreen (Iiyama T1931SR-B1, 19″, 1280×1024 pixels, ISO 5 ms) encased in a custom-built setup (see Kret et al., 2016 for further details). Bonobos were rewarded through an automated feeder system that dropped small apple cubes in their enclosure after touching the probe in the dot-probe task. Bonobos were filmed during the study to ensure correct execution of the trials (i.e., that they attended to the screen during stimulus presentation and were not interrupted or distracted by group members and to ensure the proper functioning of the equipment).

#### Procedure

As bonobos recently took part in another dot-probe study, they did not require any training. For bonobos, a trial started with a black dot presented on the lower middle part of the screen (i.e., the “start dot,” replacing the fixation cross used in the human study), which they then had to touch to start the trial. Upon touching this dot, two stimuli immediately appeared on the left and right side of the screen and remained visible for 300 ms. As in Experiment 1, the two stimuli were always of the same individual, but with one stimulus depicting large pupils and the other small pupils. Bonobos never saw stimuli with pictures of themselves. Stimulus presentation was followed by the presentation of another black dot (the probe) replacing one of the two stimuli. Upon correctly touching the probe, the bonobos automatically received a food reward. The next trial (again starting with the black start dot) started after 2,000 ms, during which a blank (white) screen was visible.

Bonobos completed 25 trials in each session. For each session, the presentation of stimuli was randomized, and the probe location was counter balanced. Erroneous trials (due to for instance bonobos not looking at the screen during stimulus presentation, not immediately touching the probe, being disturbed by another individual, or due to the touch not immediately being registered by the screen, or another individual completing the trial) were detected using the video recordings, and each trial was coded by two experts who showed good reliability (ICC = 0.827). This resulted in a total of 381 trials (*M* = 95.25, *SD* = 12.92), of which 194 were congruent and 187 were incongruent.

#### Statistical Analysis

We used linear mixed multilevel models to examine the effect of pupil size on reaction times in a dot-probe task. Trials were coded as “Congruent” when the probe appeared on the side on which the large pupils were displayed and coded as “Incongruent” when the probe appeared in the position of the small pupils. Trials were nested within subjects. We added random intercepts for subjects and for “order of trials.” Because this model did not converge, the random intercept for “order of trials” was removed. This resulted in the main model: Reaction time ~ Pupil size + (1|subject). This model was subsequently compared with a null model (that only contained the random effects) based on AIC scores (Akaike, 1974; Symonds & Moussalli, 2011).

All trials in which an incorrect response was given (i.e., when the bonobo touched the wrong side of the screen) were excluded. All reaction times below 200 ms and above 5,000 ms were excluded by default. Subsequently, all reaction times that deviated from the mean by more than 2.5 times the median absolute deviation (MAD) were excluded (Leys et al., 2013). This MAD filtering was done per participant. See Table 3 for an overview of the number of included trials.

**Table 3:** Number of included trials: Experiment 2

Congruency	Congruent	Incongruent
Number of included trials per participant	Mean: 40	Mean: 41
	Min: 21	Min: 26
	Max: 50	Max: 55
Percentage of trials excluded per participant	Mean: 19.1	Mean: 13.2
	Min: 8.3	Min: 2.3
	Max: 48.8	Max: 36.6

The statistical analysis was performed in R (R Core Team, 2020), using the “lme4” package (Bates et al., 2015).

### Results: Experiment 2

**Fig. 3:**
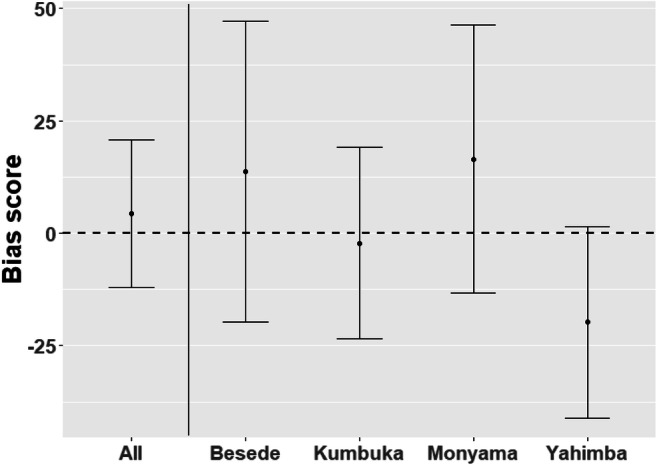
Bias score for all four bonobo participants together as well as separately. The *y*-axis shows the difference in reaction time between congruent and incongruent trials (i.e., trials in which the probe replaced large pupils and small pupils respectively). A larger bias score reflects that reaction times in the congruent trials were faster. Bias scores are in milliseconds. The bars indicate a 95% confidence interval around the estimate

To investigate an attentional bias towards pupil size in bonobos, we constructed a multilevel model containing pupil size as a fixed effect and subject ID as a random effect. This model was not a significantly better fit than the null model that contained the same random effects but not the fixed effects (delta AIC = 1.8). See supplements for an overview of this model comparison (Table ESM2), a summary of the constructed model (Table ESM3), and a summary of the results (Table ESM6 and Table ESM7) on an exploratory analysis of whether familiarity with the bonobos on the stimuli (i.e., familiar or unfamiliar) modulated a potential bias towards pupil size. Figure 3 shows a bias score towards large pupils compared to small pupils for all bonobo participants together as well as individual bias scores.

Our method of analysis cannot tell us anything about whether our effect of interest is indeed not present, or whether our sample size is simply insufficient to draw conclusions. To substantiate null findings, the calculation of a Bayes Factor (BF) can be useful (Hoijtink et al., 2019). The BF quantifies the amount of evidence for or against a hypothesis, including the null hypothesis, making it possible to draw conclusions on which hypothesis better predicts our results (Keysers et al., 2020). To calculate a BF, we first fitted a Bayesian mixed model using a Gaussian distribution, with pupil size as a fixed effect and ID as a random effect. We used a conservative Gaussian prior for the intercept (*M* = 0, *SD* = 2) as well as for the fixed effect (*M* = 0, *SD* = 1). For the random effect, we used the default half Student-*t* prior with 3 degrees of freedom. We also fitted an intercept-only (or: null) model with the same parameters (but excluding the fixed effect and its prior). Both models were run with 4 chains and 10,000 iterations, of which 1,000 were warmup iterations. Model convergence was inspected by inspecting the trace plots and histograms of the posteriors (Depaoli & van de Schoot, 2017). No divergences were found. These analyses were performed using the package “brms” (Bürkner, 2017, 2018). The same package was used to subsequently calculate a BF. To get insight into the reliability of this BF, we calculated an average BF over 100 samples, and found a BF01 = 0.993 (*SD* = 0.023). This BF indicates no clear preference for our null or alternative hypothesis.

### Conclusion: Experiment 2

The current experiment did not find evidence supporting an attentional bias towards pupil size in bonobos. Results from an alternative analysis using a Bayesian framework also did not provide evidence for either the null or the alternative hypothesis.

## Discussion

Being able to quickly and accurately estimate the emotional state of a group member is an essential skill for both bonobos and humans. They are able to do this, in part thanks to an attentional bias towards expressions of emotion and social or emotional scenes (Phelps et al., 2006; Schupp et al., 2003; Vuilleumier, 2005). Several studies suggest that eyes, and in particular changes in pupil size, can give reliable information about the arousal state of others. However, whether the aforementioned attentional bias towards emotions extends to an unconsciously perceived and produced signal such as changes in pupil size had not yet been investigated. In two dot-probe experiments, one in humans and one in bonobos, this study investigated whether both species attention is modulated by the pupil size of the observed. In humans, a small but significant bias towards large pupils was found when compared to intermediate-sized pupils. Similarly, humans showed an attentional bias towards intermediate-sized pupils when compared to small pupils. In contrast, bonobos showed no significant attentional bias.

As expected, Experiment 1 indicated that pupil size can modulate attention in humans. Interestingly, the prediction that both large and small pupils would be perceived as socially relevant signals and would therefore attract more attention when compared to intermediate-sized pupils was not supported by our findings. Our findings suggest that humans have an attentional bias towards larger pupils in general. Since pupil dilation can indicate increased emotional arousal as a response to seeing both negative and positive stimuli (Bradley et al., 2008), larger pupils may simply be more biologically salient than small pupils. Especially during eye contact as is mostly the case during social interactions, pupil dilation may signal social interest. Pupil constriction, on the other hand, may reflect fatigue or boredom and could signal the end of an interaction, resulting in the motivation to direct attention elsewhere. As such, an attentional bias towards larger pupils may function to rapidly detect arousal in others, which is a salient signal that can help the observer predict others’ behaviors and intentions. Whether arousal indicates a fleeting opportunity to gain something positive or rather an arousing negative event, in both cases the quick detection of dilated pupils, as indicators of heightened arousal in others, would allow the observer to form an appropriate response that can subsequently increase survival chances (Öhman et al., 2001). Future work could aim to investigate the effects that different contexts may have on how small and large pupil sizes are being perceived and how that perception modulates attentional processes.

Contrary to our expectations, we did not observe an attentional bias towards either large or small pupils in bonobos. One interpretation is that bonobos indeed lack this bias. Evidence from the field of comparative morphology support this notion. Bonobos seem to have dark irises that make it more difficult to distinguish the pupil (Perea-García et al., 2019), while humans display a range of combinations of different hues and brightness of the iris (Negro et al., 2017). Since pupils are invariably black, while iridal brightness varies across a continuum (Perea García et al., 2017), we assume that variation in iridal brightness is one of the main factors affecting the visibility of pupils. Interestingly, chimpanzees have bright irises (Caspar et al., 2021) that have been suggested to facilitate the perception of changes in pupil size by observers (Perea-García et al., 2021). Indeed, previous studies show that pupil size changes are perceived by chimpanzees (Kret et al., 2014), suggesting communicative functions for the perception of changes in pupil size in the Pan lineage. The apparent lack of specialization in bonobo eyes to facilitate the perception of pupil size changes by observers would be expected in a species that does not rely on such cues in their social interactions. Conversely, the conspicuous appearance of chimpanzee pupils suggests that the perception of changes in pupil size is part of their repertoire of social cues.

Indeed, a more likely explanation for our null findings in bonobos is our limited sample size. For example, if we take the individual differences observed in our bonobo participants (see Fig. 3) at face value, it is easy to imagine that a single non-typical individual (e.g., Yahimba) might influence the overall results to a much larger extent than a single outlier in a sample of 150 humans. To support this idea, a Bayesian statistical analysis was performed to investigate whether our data provided more support for the alternative hypothesis or the null hypothesis. The results of this analysis were inconclusive. As the absence of an effect does not mean there really is no effect, and our Bayesian analysis provided inconclusive results, the results of this study do not justify a claim in either direction. As such, the most obvious way forward is to repeat this experiment with an increased sample size, thereby increasing statistical power and making it possible to generalize findings. There were several other ways in which the two experiments differed apart from sample size, which may have affected the results. Most notably, the human experiment always presented an intermediate-sized pupil next to either a large or a small pupil, while the bonobo experiment always presented large and small pupils next to each other. However, considering that in humans, there appears to be a general bias towards larger pupils in either comparison, this difference in pupil size comparisons should not have negatively impacted our ability to find this same effect in bonobos, if it truly exists. However, if bonobos, unlike humans, would have an attentional bias towards both larger and smaller pupils when compared to intermediate-sized pupils, the current method would have been inadequate to measure these biases. Furthermore, using images of faces in the bonobo experiment rather than images of only the eye region, as in the human experiment, may have obscured the differences in pupil sizes between the competing images, by dampening the overall attention to the eye region. While it could be argued that these images are more naturalistic compared to viewing just the eye region, it may have also resulted in making an already subtle cue too small to notice quickly. Finally, a recent study exploring attention to familiar and unfamiliar faces in bonobos and chimpanzees (Lewis et al., 2021) showed that familiarity modulates attention. In line with this, Van Berlo et al. (2020) found that familiarity and emotional expressions have an interactive effect on attention. Specifically, bonobos appeared to have a bias towards emotions from unfamiliar individuals but not familiar ones. Our exploratory analysis did not reveal a modulating effect of familiarity on a bias for pupil size, but given that this factor was not a part of our initial research question, our picture set may have been too unbalanced to uncover such an effect as we had more pictures depicting familiar individuals. To address these issues, this study should be repeated in bonobos, using a different set of stimuli, specifically made to match the one used in humans more closely, that is, full color images that are zoomed in and cropped to only show the eye region of, preferably, unfamiliar bonobos. Alternatively, future studies could look deeper into effects of pupil size on attention comparing different types of stimuli (i.e., faces versus eyes only), and potential interactions between attention for larger pupils and familiarity.

Another difference between the two experiments is that the human experiment was conducted online, without direct supervision. For instance, while making sure to reduce possible sources of distraction was emphasized before the start of the experiment, it was not possible to control and supervise to ensure proper participation. We controlled for this potential confound by filtering our data based on the median absolute deviation, and by removing trials in which participants responded by pressing the wrong button as well as excluding trials in which reaction times were either improbably fast or very slow, since this suggested that the participant was either simply pressing the keys rapidly without paying attention or that they had been distracted during stimulus presentation. Although it is not possible to know to what extent, this factor may have introduced at least some additional variation in the human experiment, subsequently contributing to a smaller effect size. However, our effect sizes are in line with previously found effect sizes using the dot-probe task to establish attentional biases towards emotions (van Rooijen et al., 2017). Although this was not physically possible at the time that this study was conducted, ideally a replication of Experiment 1 would be presented to human participants in a situation in which it is possible to monitor the behavior of the participant, similarly to Experiment 2.

To conclude, our study shows that in humans, attention is prioritized towards larger pupils, possibly reflecting a rapid detection of arousal in others. We did not find this bias in bonobos. Possibly, the differences in eye morphology may have contributed to the development of different attentional prioritizations in humans and bonobos, but more likely, methodological constraints may have contributed to our null findings, making it difficult to draw a clear conclusion for bonobos. Nevertheless, our findings suggest that the attentional bias that humans have towards emotions in general extends to a subtle, unconsciously perceived and produced signal, namely pupil dilation. This result contributes to a small but growing body of literature emphasizing the social signalling function of eyes, and in particular pupil size.

## Supplementary information

ESM 1 (PDF 164 kb)

ESM 2 (XLSX 21 kb)

## Additional Information

### Data Availability

All data and materials associated with this article can be found here: 10.34894/HABM7O.

### Code Availability

All software and code adjustments that are associated with this paper will, after publication of the manuscript, be uploaded and made openly accessible on the archiving platform DataverseNL through the last author’s institute, Leiden University.
