# Supplementary material for: Attention Towards Pupil Size in Humans and Bonobos (Pan paniscus)
Source: Affect Sci. 2022 Nov 11;3(4):761–71. doi: 10.1007/s42761-022-00146-1 (PMC9743857; doi:10.1007/s42761-022-00146-1)
Supplement: Supplementary file 1 — (PDF 164 kb) [file 42761_2022_146_MOESM1_ESM.pdf]

# Attention towards pupil size in humans and bonobos (*Pan Paniscus*)

Zijlstra, T. W.[1,2], van Berlo, E.[1,2,3] & Kret, M. E,[1,2].

1. Cognitive Psychology Unit, Institute of Psychology, Leiden University, Leiden, the Netherlands
2. Leiden Institute for Brain and Cognition (LIBC), Leiden, the Netherlands
3. Institute for Biodiversity and Ecosystem Dynamics, University of Amsterdam, Amsterdam, the Netherlands

**corresponding author**: t.w.zijlstra@fsw.leidenuniv.nl

**ORCID:**

Zijlstra, T.W.: 0000-0001-6271-6141

Van Berlo, E.: 0000-0002-5523-7721

Kret, M.E.: 0000-0002-3197-5084

**Keywords**: pupil, attention, eyes, imitation, bonobos, sociality

## Statements and Declarations

The authors have no relevant financial or non-financial interests to disclose.

**Supplementary material**

*Null-model comparisons*

Table ESM2: Experiment 1, Humans, null-model comparison

| | npar | AIC | BIC | logLik | deviance | Chisquare | Df | p |
|---|---|---|---|---|---|---|---|---|
| model0 | 5 | 251977 | 252018 | -125984 | 251967 | | | |
| model1 | 8 | 251965 | 252030 | -125975 | 251949 | 18.183 | 3 | <0.001 |

model0: RT ~ (1 | Subject) + (1 | Trial) + (1 | Stimulus)

model1: RT~ Pupil size * Congruency + (1 | Subject) + (1 | Trial) + (1 | Stimulus)

Table ESM2: Experiment 2, Bonobos, null model comparison

| | npar | AIC | BIC | logLik | deviance | Chisquare | Df | p |
|---|---|---|---|---|---|---|---|---|
| model0 | 3 | 3743.2 | 3754.6 | -1868.6 | 3737.2 | | | |
| model1 | 4 | 3745.0 | 3760.1 | -1868.5 | 3737.0 | 0.2706 | 1 | 0.6029 |

model0: RT ~ (1 | Subject)

model1: RT ~ Pupil size + (1 | Subject)

*Model summary*

Table ESM3: Experiment 2, Bonobos, model summary

| Fixed effects | Estimate | Std. Error | T value | p | Cohen's d |
|---|---|---|---|---|---|
| Pupilsize (small) | 4.350 | 8.356 | 0.521 | 0.60 | 0.031 |

*Exploratory analysis: Impact of familiarity*

Table ESM6: Experiment 2, Bonobos, exploratory analysis – null model comparison

| | npar | AIC | BIC | logLik | deviance | Chisquare | Df | p |
|---|---|---|---|---|---|---|---|---|
| model0 | 3 | 3743.2 | 3754.6 | -1868.6 | 3737.2 | | | |
| model1 | 6 | 3747.4 | 3770.1 | -1867.7 | 3735.4 | 1.8344 | 3 | 0.6075 |

model0: RT ~ (1 | Subject)

model1: RT~ Pupil size * Group + (1 | Subject)

Table ESM7: Experiment 2, Bonobos, exploratory analysis - model summary

| Fixed effects | Estimate | Std. Error | T value | p | Cohen's d |
|---|---|---|---|---|---|
| Pupilsize (small) | 10.327 | 9.644 | 1.071 | 0.2851 | 0.074 |
| Group (unfamiliar) | 12.515 | 14.257 | 0.878 | 0.3807 | 0.089 |
| Pupilsize * Group | -24.234 | 19.475 | -1.244 | 0.2143 | 0.172 |

*Examples of Bonobo stimuli*

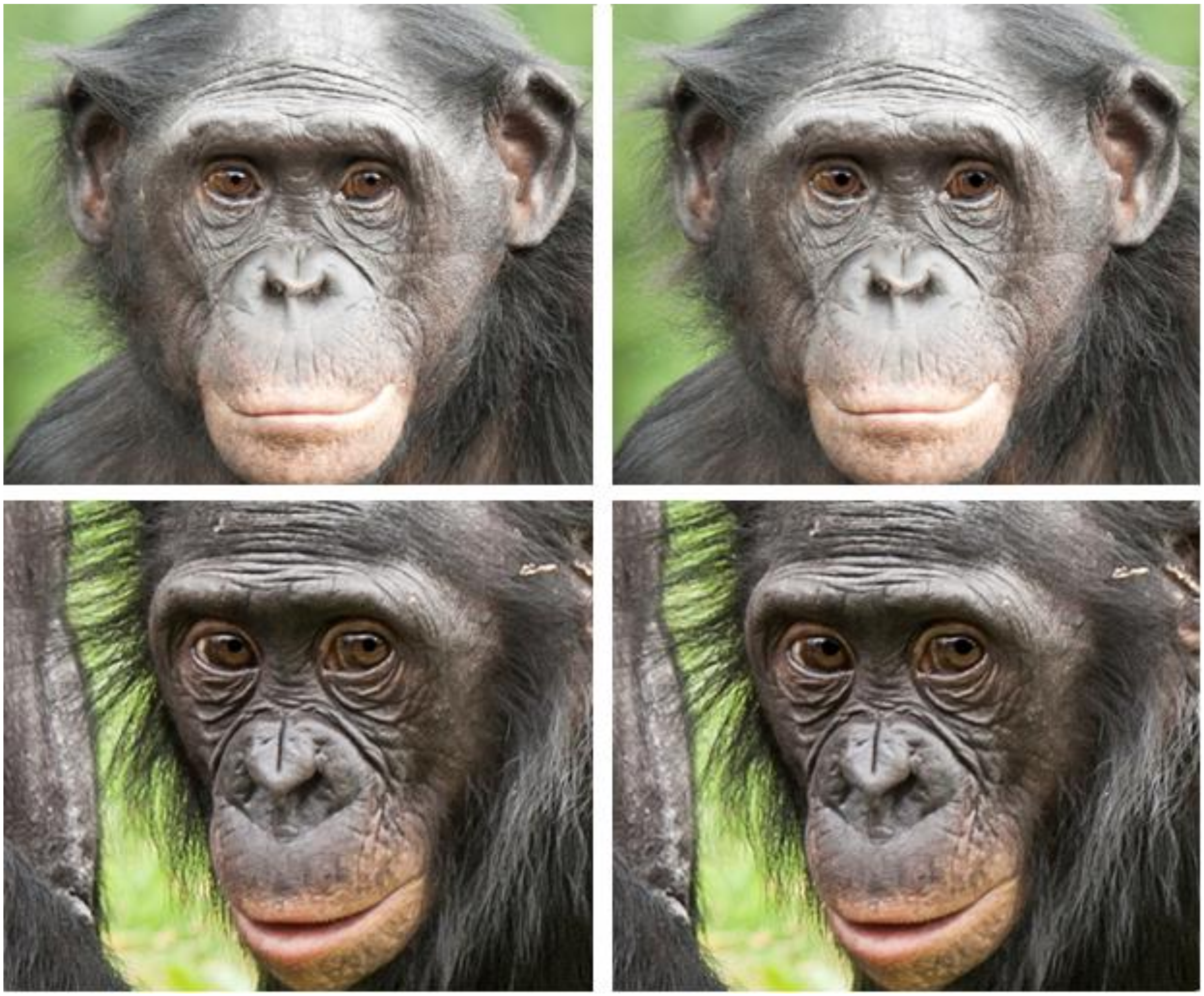

Figure ESM1. Two examples of the bonobo stimulus set, with small pupils on the left, and large pupils on the right.
